# Supplementary material for: Quantitative contribution of the spacer length in the supercoiling-sensitivity of bacterial promoters
Source: Nucleic Acids Res. 2022 Jul 1;50(13):7287–97. doi: 10.1093/nar/gkac579 (PMC9303308; doi:10.1093/nar/gkac579)
Supplement: gkac579_Supplemental_File [file gkac579_supplemental_file.pdf]

Supplementary Information for

Quantitative contribution of the spacer length in the supercoiling-sensitivity of bacterial promoters

Raphaël Forquet, William Nasser, Sylvie Reverchon and Sam Meyer*

Université de Lyon, INSA-Lyon, Université Claude Bernard Lyon 1, CNRS, UMR5240 MAP, F-69622, France

* To whom correspondence should be addressed. Email: sam.meyer@insa-lyon.fr.

June 14, 2022

This PDF file includes:

- Supplementary text
- Supplementary figures S1 to S7
- Supplementary tables S1 to S2
- Supplementary information references

Supplementary text

Supplementary equations (additional model computations)

The prediction of relative *in vitro* expression levels of mutant spacers (Eq. 4) simplifies, after linear expansion in $\Delta n/n_0 = (n - n_0)/n_0$, as:

$$\log\left(\frac{k(\sigma, n_0 + \Delta n, s)}{k(\sigma, n_0, s)}\right) \simeq Q_{sp}(n, s) - \alpha_0 k_\theta \Delta n \left[\frac{\theta_P}{n_0}\left(1 - \frac{1}{2}\frac{\Delta n}{n_0}\right) - (1+\sigma)\alpha_0\right] \quad \textbf{(S1)}$$

where the dependency in σ (slope) is defined without any adjustable parameter, and is proportional to k_θ (torsional stiffness of DNA) and to Δn.

The relative *in vivo* effect of a superhelical variation $\sigma_0 \to \sigma_0 + \Delta\sigma$ on a promoter s (log fold-change) is predicted as:

$$\text{logFC}(\Delta\sigma, \sigma_0, n, s) = -\frac{n}{2} k_\theta \left[\alpha_0^2 \left(\Delta\sigma^2 + 2\Delta\sigma(1+\sigma_0)\right)\right] - \left[k_\theta\, \theta_P\, \alpha_0\, \Delta\sigma + R_{sc}(\Delta\sigma, \sigma_0, s)\right] \quad \textbf{(S2)}$$

where $R_{sc} = \Delta G_{sc}(\sigma_0 + \Delta\sigma, s) - \Delta G_{sc}(\sigma_0, s)$ reflects all spacer length-independent regulatory effects of SC on the considered promoter. Crucially, the spacer length-dependent term does not depend on any unknown parameter. A linear expansion in $\Delta\sigma$ results in the entirely predictable Eq. 5.

Genome-wide analyses of spacer responses to supercoiling variations in distant bacteria

Transcriptomic data were collected from the literature (Supplementary Tab. S2). Since curated promoter maps were not available, genome-wide TSS maps and associated genes were retrieved from literature (Supplementary Tab. S2), and a scan for promoter motifs upstream of TSSs was conducted with bTSSfinder [1]. Only σ70-dependent promoters were retained for all organisms, except for the cyanobacterium *S. elongatus*, for which only σA-dependent promoters were kept (primary σ factor), and classified depending on their spacer length and response to the investigated condition. The thresholds for statistical selection procedures are indicated in Supplementary Tab. S2, and were adjusted to generate subsets of act/rep genes of sizes comparable among the different data sets, while having enough statistical power for the analysis. The relation between promoter activation and spacer length was then quantified by a Student's t-test between activated and repressed promoters (Supplementary Fig. S4), such as in Fig. 4 and 5. All error bars shown are 95% confidence intervals.

Modelling of the effect of spacer sequence on -35/-10 alignment by RNA Polymerase

To compute the contribution of the spacer sequence in the model (Discussion), we included the sequence-dependence of two parameters of Eq. 1, the twist angle (α_0) and the torsional stiffness (k_θ), based on the parameters of the rigid base-pair model inferred from high-resolution crystallographic structures of DNA oligomers [2] and implemented in TwistDNA [3]. Based on these parameters, Eq. 1 was modified to include the total angle between -35/-10 binding sites and the total spacer stiffness, resulting in the following:

$$\Delta G(\sigma, s) = \frac{1}{2} k_\theta(s) \left(\theta_P - \theta_0(s) - \alpha_0 \frac{k_\theta}{k_\theta(s)} \sigma \right)^2$$

where σ is the supercoiling level, s is the promoter spacer sequence, $k_\theta(s)$ is the DNA spacer twist stiffness estimated with ThreaDNA [3], $\theta_P = 543$ is the optimal angle between -35 and -10 sites for RNAP binding, as assumed in the main text (with most predictions given in Discussion being independent of its value), $\theta_0(s)$ is the effective angle estimated with ThreaDNA [3] which depends on base composition, $\alpha_0 = 34°$ is the average twist angle between adjacent nucleotides, and k_θ is the average (base-pair step) twist stiffness. Then, for each *E. coli* σ70-dependent promoter, the response to DNA relaxation was predicted starting from a level $\sigma = -0.06$, with a relaxation magnitude $\Delta\sigma = 0.03$ (Supplementary Fig. S7).

Supplementary figures

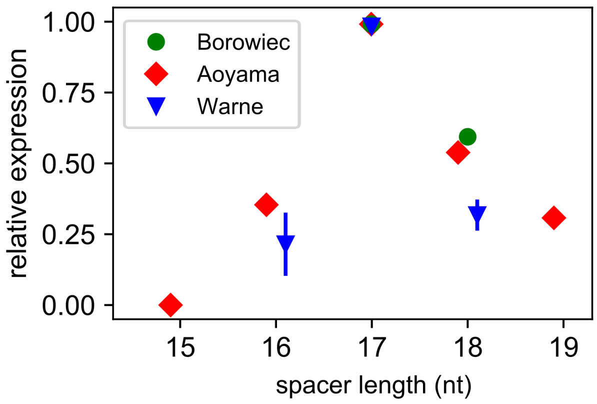

Supplementary Figure S1: Relative *in vitro* promoter expression levels depending on spacer length, for a supercoiling level $\sigma = -0.06$, using data from Borowiec et *al.* [4] (green), Aoyama et *al.* [5] (red), and Warne et *al.* [6] (blue). Error bars are shown for [6] due to the use of different promoters. All expression levels are normalised by those of the corresponding promoter containing a 17-nt spacer.

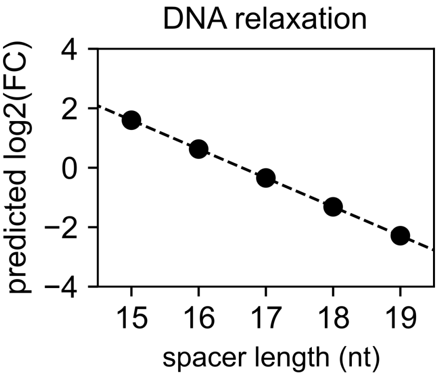

Supplementary Figure S2: Predicted expression fold-changes for promoters containing 15 to 19-nt spacers, during a relaxation from $\sigma = -0.06$ to $\sigma = -0.03$.

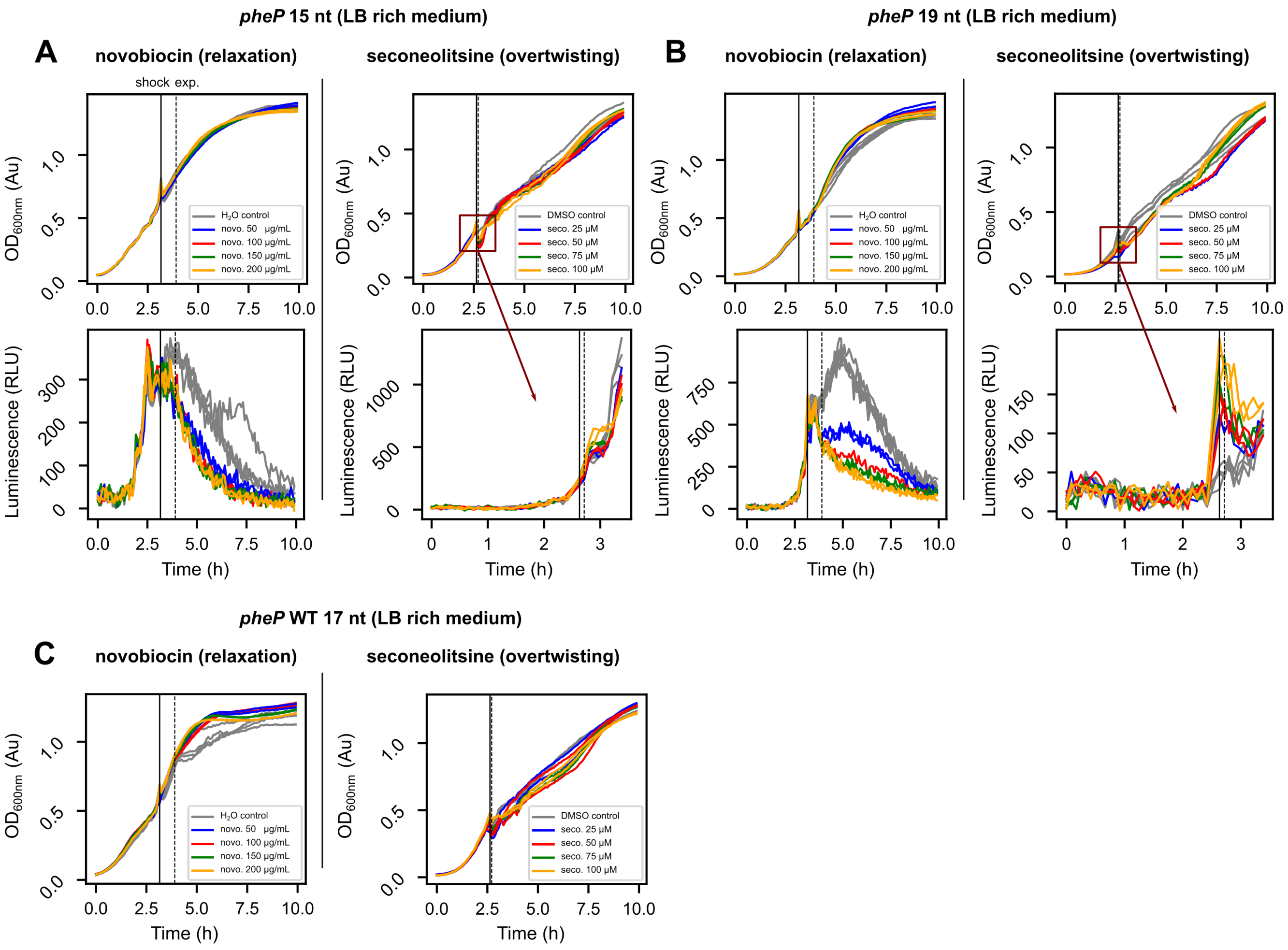

Supplementary Figure S3: Bacterial growth and promoter expression monitored in a microplate reader (raw data). **(A)** *E. coli* bacteria carrying plasmids with the *pheP* mutant promoter with a 15-nt spacer. **(B)** *pheP* mutant promoter with a 19-nt spacer. **(C)** *pheP* native promoter containing a 17-nt spacer. For all promoters, upper panels show the bacterial growth measured by OD_{600nm}, lower panels the expression levels measured by luminescence, left panels the experiments conducted with novobiocin, and right panels the experiments conducted with seconeolitsine. "Shock" and "exp." refer to the time points used for the antibiotic shock, and for the computation of expression fold-changes, respectively. Due to the stronger and more transient expression variation induced by seconeolitsine shock, a zoom is performed for luminescence. Some OD_{600nm} curves exhibit slight discrepancies at shock time or after several hours; these are optical artefacts due to the opening of the recorder and/or the formation of sediments disrupting the measurements. The luminescence curves of *pheP* native 17-nt spacer promoter are provided in Fig. 3.

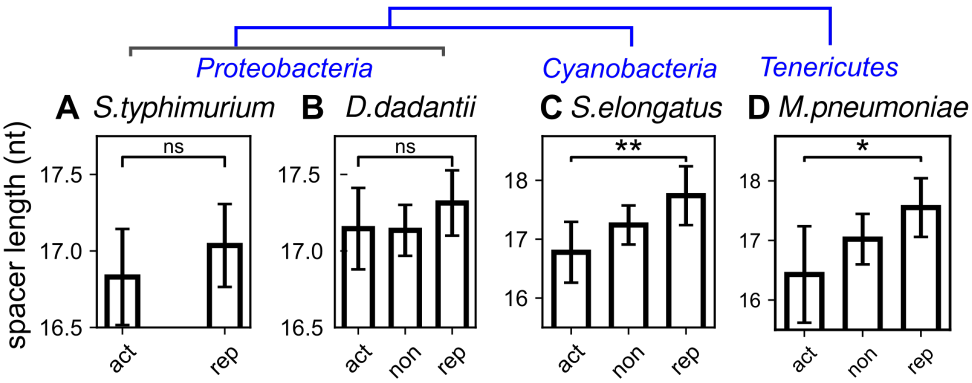

Supplementary Figure S4: Statistical relation between spacer length and promoter response to DNA relaxation (act: activated, non: not significantly affected, rep: repressed) in distant bacterial species (see Supplementary Tab. S2 for experimental conditions and references): **(A)** *Salmonella typhimurium* ($P = 0.169$), **(B)** *Dickeya dadantii* ($P = 0.170$), **(C)** *Synechococcus elongatus* ($P = 0.007$), **(D)** *Mycoplasma pneumoniae* ($P = 0.017$). In proteobacteria and *M. pneumoniae*, only $\sigma 70$ promoters were considered, whereas only σA promoters were considered for *S. elongatus*. A schematic phylogeny is depicted above.

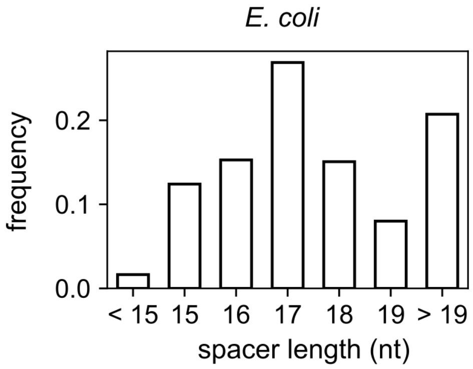

Supplementary Figure S5: Distribution of spacer lengths at *E. coli* $\sigma 70$-dependent promoters, based on the Ecocyc database [7].

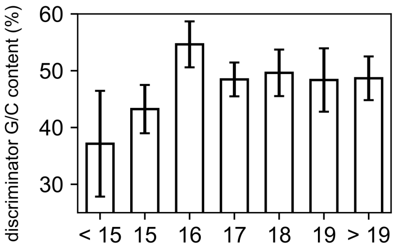

Supplementary Figure S6: Analysis of the coupling between promoter spacer length and discriminator G/C-content, based on the Ecocyc database [7]. Promoters with 16-nt spacers exhibit G/C-richer discriminators compared to other promoters (P-value < 0.001), 15-nt and < 15-nt spacers exhibit A/T-richer discriminators (P-value = 0.015 and 0.004, respectively), whereas 17, 18, 19 and > 19-nt spacers have similar, not significantly different discriminator A/T-contents.

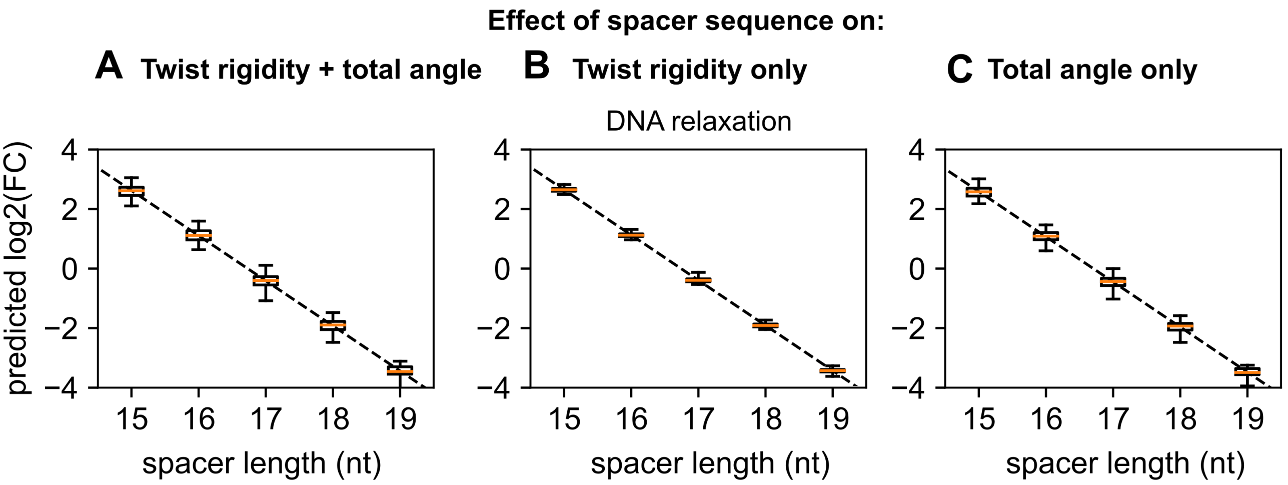

Supplementary Figure S7: Effect of the spacer sequences: boxplots show the span of predicted fold-changes for all $\sigma 70$-dependent promoters of *E. coli* (based on the Ecocyc database [7]), during a relaxation shock from $\sigma = -0.06$ to $\sigma = -0.03$. The box extends from the first quartile to the third quartile values of the data, with an orange line at the median, and with whiskers extending from each end of the box to the extreme values. **(A)** With sequence-dependent values for both twist angle and rigidity: the maximal magnitude of the sequence effect is comparable to that of a gain or loss of one nucleotide in the spacer. **(B)** With sequence-dependent spacer twist rigidity and sequence-averaged total angle, the effect of the sequence is strongly reduced, showing that the latter mostly modulates the angle. **(C)** With sequence-dependent total angle and sequence-averaged spacer twist rigidity.

Supplementary tables

Promoter	Sequence
>pheP_WT_17nt	CTCGAGTCAGAGGTGATGAGCCGGATTGCCGCGCCGATGATTGGCGGCATGATCACCGCACCTTTGCTGTCGCTGTTTATT<br>ATCCCGGCGGCGTATAAGCTGATGTGGCTGCACCGACATCGGGTACGGAAATAAAAGCAGGATACCCCGTTTAACCGTGTG<br>GATTGTGTCTTGCGA**CGATGGGCACTAAATGT**TAAAAGGTGCCCCTCAACAAAAAAGACACACAGGGGAAAGGCGGATCC
>pheP_15nt	CTCGAGTCAGAGGTGATGAGCCGGATTGCCGCGCCGATGATTGGCGGCATGATCACCGCACCTTTGCTGTCGCTGTTTATT<br>ATCCCGGCGGCGTATAAGCTGATGTGGCTGCACCGACATCGGGTACGGAAATAAAAGCAGGATACCCCGTTTAACCGTGTG<br>GATTGTGTCTTGCGA**CGATGGGCTAAATGT**TAAAAGGTGCCCCTCAACAAAAAAGACACACAGGGGAAAGGCGGATCC
>pheP_19nt	CTCGAGTCAGAGGTGATGAGCCGGATTGCCGCGCCGATGATTGGCGGCATGATCACCGCACCTTTGCTGTCGCTGTTTATT<br>ATCCCGGCGGCGTATAAGCTGATGTGGCTGCACCGACATCGGGTACGGAAATAAAAGCAGGATACCCCGTTTAACCGTGTG<br>GATTGTGTCTTGCGA**CGATACGGGCACTAAATGT**TAAAAGGTGCCCCTCAACAAAAAAGACACACAGGGGAAAGGCGGATCC
	XhoI restriction site, -35 element, **spacer**, -10 element, TSS, BglII restriction site

Plasmid	Description	Origin
pUCTer-*luc*	High-copy-number vector (pUC18 derivative) containing a multiple cloning site upstream of the *luc* reporter gene, followed by a *rrnB* terminator and a *cat* gene conferring chloramphenicol resistance.	Laboratory collection

Supplementary Table S1: List of *pheP*-derived synthetic promoter sequences with mutated spacers, and pUCTer-*luc* plasmid used in this study.

Species	Condition	Threshold adj. P-value	Threshold $\log_2$(FC)	Supercoiling variation	Transcription start sites reference
Escherichia coli	norfloxacin [8]	0.05	0	relaxation	[7]
	inheritable supercoiling variation [9]	0.1	0	overtwisting	[7]
Salmonella typhimurium	*gyrA* mutant [10]	NA	0.4	relaxation	[11]
Dickeya dadantii	novobiocin [12]	0.05	0	relaxation	[13]
Synechococcus elongatus	supercoiling correlation* [14]	NA	0.4	relaxation/ overtwisting	[15]
Mycoplasma pneumoniae	novobiocin [16]	0.1	0	relaxation	[16]

Supplementary Table S2: Compilation of investigated species, conditions and references. The thresholds for statistical selection procedures are indicated, and were adjusted to generate subsets of activated/repressed genes of sizes comparable among the different data sets. For a threshold of 0.4 on the $\log_2$(fold-change), genes are considered activated for a $\log_2(FC) > 0.4$, repressed for a $\log_2(FC) < -0.4$, and not significantly affected for a $\log_2$(FC) comprised between -0.4 and 0.4. The correlation* condition from *S. elongatus* corresponds to the phasing of gene expression in the SC circadian oscillation and provides an indirect proxy of gene response to SC relaxation [14].

References

[1] Shahmuradov, I. A., Mohamad Razali, R., Bougouffa, S., Radovanovic, A., and Bajic, V. B. (2017) bTSSfinder: a novel tool for the prediction of promoters in cyanobacteria and Escherichia coli. *Bioinformatics,* **33**(3), 334–340.

[2] Xu, F. and Olson, W. K. (2010) DNA architecture, deformability, and nucleosome positioning. *Journal of Biomolecular Structure & Dynamics,* **27**(6), 725–739.

[3] Cevost, J., Vaillant, C., and Meyer, S. (2018) ThreaDNA: predicting DNA mechanics' contribution to sequence selectivity of proteins along whole genomes. *Bioinformatics,* **34**(4), 609–616.

[4] Borowiec, J. A. and Gralla, J. D. (1987) All three elements of the lac ps promoter mediate its transcriptional response to DNA supercoiling. *Journal of Molecular Biology,* **195**(1), 89–97.

[5] Aoyama, T. and Takanami, M. (1988) Supercoiling response of E. coli promoters with different spacer lengths. *Biochimica Et Biophysica Acta,* **949**(3), 311–317.

[6] Warne, S. E. and deHaseth, P. L. (1993) Promoter recognition by Escherichia coli RNA polymerase. Effects of single base pair deletions and insertions in the spacer DNA separating the -10 and -35 regions are dependent on spacer DNA sequence. *Biochemistry,* **32**(24), 6134–6140.

[7] Keseler, I. M., Mackie, A., Santos-Zavaleta, A., Billington, R., Bonavides-Martínez, C., Caspi, R., Fulcher, C., Gama-Castro, S., Kothari, A., Krummenacker, M., Latendresse, M., Muñiz-Rascado, L., Ong, Q., Paley, S., Peralta-Gil, M., Subhraveti, P., Velázquez-Ramírez, D. A., Weaver, D., Collado-Vides, J., Paulsen, I., and Karp, P. D. (2017) The EcoCyc database: reflecting new knowledge about Escherichia coli K-12. *Nucleic Acids Research,* **45**(Database issue), D543–D550.

[8] Blot, N., Mavathur, R., Geertz, M., Travers, A., and Muskhelishvili, G. (2006) Homeostatic regulation of supercoiling sensitivity coordinates transcription of the bacterial genome. *EMBO Reports,* **7**(7), 710–715.

[9] El Houdaigui, B., Forquet, R., Hindré, T., Schneider, D., Nasser, W., Reverchon, S., and Meyer, S. (2019) Bacterial genome architecture shapes global transcriptional regulation by DNA supercoiling. *Nucleic Acids Research,* **47**(11), 5648–5657.

[10] Webber, M. A., Ricci, V., Whitehead, R., Patel, M., Fookes, M., Ivens, A., and Piddock, L. J. V. (2013) Clinically relevant mutant DNA gyrase alters supercoiling, changes the transcriptome, and confers multidrug resistance. *mBio,* **4**(4), e00273–13.

[11] Kröger, C., Dillon, S. C., Cameron, A. D. S., Papenfort, K., Sivasankaran, S. K., Hokamp, K., Chao, Y., Sittka, A., Hébrard, M., Händler, K., Colgan, A., Leekitcharoenphon, P., Langridge, G. C., Lohan, A. J., Loftus, B., Lucchini, S., Ussery, D. W., Dorman, C. J., Thomson, N. R., Vogel, J., and Hinton, J. C. D. (2012) The transcriptional landscape and small RNAs of Salmonella enterica serovar Typhimurium. *Proceedings of the National Academy of Sciences of the United States of America,* **109**(20), E1277–E1286.

[12] Jiang, X., Sobetzko, P., Nasser, W., Reverchon, S., and Muskhelishvili, G. (2015) Chromosomal "Stress-Response" Domains Govern the Spatiotemporal Expression of the Bacterial Virulence Program. *mBio,* **6**(3), e00353–15.

[13] Forquet, R., Jiang, X., Nasser, W., Hommais, F., Reverchon, S., and Meyer, S. (2022) Mapping the Complex Transcriptional Landscape of the Phytopathogenic Bacterium Dickeya dadantii. *mBio,* p. e0052422.

[14] Vijayan, V., Zuzow, R., and O'Shea, E. K. (2009) Oscillations in supercoiling drive circadian gene expression in cyanobacteria. *Proceedings of the National Academy of Sciences of the United States of America,* **106**(52), 22564–22568.

[15] Vijayan, V., Jain, I. H., and O'Shea, E. K. (2011) A high resolution map of a cyanobacterial transcriptome. *Genome Biology,* **12**(5), R47.

[16] Junier, I., Unal, E. B., Yus, E., Lloréns-Rico, V., and Serrano, L. (2016) Insights into the Mechanisms of Basal Coordination of Transcription Using a Genome-Reduced Bacterium. *Cell Systems,* **2**(6), 391–401.
